# Supplementary material for: Non-invasive single-cell morphometry in living bacterial biofilms
Source: Nat Commun. 2020 Dec 1;11:6151. doi: 10.1038/s41467-020-19866-8 (PMC7708432; doi:10.1038/s41467-020-19866-8)
Supplement: Supplementary file 4 — Description of Additional Supplementary Files [file 41467_2020_19866_MOESM4_ESM.pdf]

Title: Supplementary Movie 1.

Description: A slice-by-slice animation the difference between the BCM3D segmentation result and the ground truth using a mixedpopulation biofilm containing spherical cells and rod-shaped cells (also see Figure S8). (Left panel) Simulated fluorescence image of a mixture of spherical cells and rod-shaped cells. Rod-shaped cells are displayed in green, and spherical cells are displayed in magenta. (Right panel) Absolute value of the difference between the BCM3D segmentation result and the ground truth. Colored pixels indicate regions where the two masks do not agree. Rod-shaped cells are displayed in green, and spherical cells are displayed in magenta.
