# Supplementary material for: Non-invasive single-cell morphometry in living bacterial biofilms
Source: Nat Commun. 2020 Dec 1;11:6151. doi: 10.1038/s41467-020-19866-8 (PMC7708432; doi:10.1038/s41467-020-19866-8)
Supplement: Supplementary file 5 — Reporting Summary [file 41467_2020_19866_MOESM5_ESM.pdf]

nature research

Corresponding author(s): Andreas Gahlmann

Last updated by author(s): Oct 22, 2020

Reporting Summary

Nature Research wishes to improve the reproducibility of the work that we publish. This form provides structure for consistency and transparency in reporting. For further information on Nature Research policies, see our Editorial Policies and the Editorial Policy Checklist.

Statistics

For all statistical analyses, confirm that the following items are present in the figure legend, table legend, main text, or Methods section.

n/a	Confirmed	
☐	☒	The exact sample size (*n*) for each experimental group/condition, given as a discrete number and unit of measurement
☐	☒	A statement on whether measurements were taken from distinct samples or whether the same sample was measured repeatedly
☒	☐	The statistical test(s) used AND whether they are one- or two-sided<br>*Only common tests should be described solely by name; describe more complex techniques in the Methods section.*
☒	☐	A description of all covariates tested
☒	☐	A description of any assumptions or corrections, such as tests of normality and adjustment for multiple comparisons
☐	☒	A full description of the statistical parameters including central tendency (e.g. means) or other basic estimates (e.g. regression coefficient) AND variation (e.g. standard deviation) or associated estimates of uncertainty (e.g. confidence intervals)
☒	☐	For null hypothesis testing, the test statistic (e.g. *F*, *t*, *r*) with confidence intervals, effect sizes, degrees of freedom and *P* value noted<br>*Give P values as exact values whenever suitable.*
☒	☐	For Bayesian analysis, information on the choice of priors and Markov chain Monte Carlo settings
☒	☐	For hierarchical and complex designs, identification of the appropriate level for tests and full reporting of outcomes
☒	☐	Estimates of effect sizes (e.g. Cohen's *d*, Pearson's *r*), indicating how they were calculated

Our web collection on statistics for biologists contains articles on many of the points above.

Software and code

Policy information about availability of computer code

Data collection	Experimental data are collected on a lattice light sheet microscope using the data acquisition program developed in LabVIEW 2013 by Coleman Technologies, Inc. Simulated data are generated with CellModeller4 (open source) and Matlab 2019.
Data analysis	3D images were rendered using the 3D Viewer plugin in Fiji 1.52p or ChimeraX v0.91.<br>The cell contours on experimental 2D slices are traced by using freehand selections in Fiji 1.52p ROI Manger.<br>Python 3.7.4 scripts written in Jupyter Notebook 6.0.1 are used for CNN data processing.<br>NiftyNet 0.6.0 is used to train CNNs.<br>The code for running all the modules of BCM3D as well as training and test data is available at https://github.com/GahlmannLab/BCM3D.git , DOI: 10.5281/zenodo.4088658

For manuscripts utilizing custom algorithms or software that are central to the research but not yet described in published literature, software must be made available to editors and reviewers. We strongly encourage code deposition in a community repository (e.g. GitHub). See the Nature Research guidelines for submitting code & software for further information.

Data

Policy information about availability of data

All manuscripts must include a data availability statement. This statement should provide the following information, where applicable:

- Accession codes, unique identifiers, or web links for publicly available datasets
- A list of figures that have associated raw data
- A description of any restrictions on data availability

Source data are provided with this paper. All data used for generating the results presented in this paper are available from the corresponding author upon request.

Field-specific reporting

Please select the one below that is the best fit for your research. If you are not sure, read the appropriate sections before making your selection.

- [x] Life sciences
- [ ] Behavioural & social sciences
- [ ] Ecological, evolutionary & environmental sciences

For a reference copy of the document with all sections, see nature.com/documents/nr-reporting-summary-flat.pdf

Life sciences study design

All studies must disclose on these points even when the disclosure is negative.

Sample size	We simulated biofilms with 5 different cell densities [48.2% to 63.2%] and 10 different Signal-to-Background ratios [1.19 to 5.27], giving 50 different conditions. In order to eliminate the bias caused by any one given cell arrangement, 10 different biofilms were simulated for each condition, so as to provide reasonable estimates of mean and standard deviation at each condition and also avoid excessive computational processing times. Time-consuming manual annotation of experimental images was performed by N = 3 researchers, to provide reasonable estimates of mean and standard deviation and also avoid excessive annotation times.
Data exclusions	No data were excluded from the analyses.
Replication	For simulated data, all attempts at replication were successful. Typically, simulated datasets were replicated N=10 times. For experimental data, findings were not replicated, because biofilm growth is a highly stochastic process and no two biofilms are alike.
Randomization	Randomization of allocating organisms into experimental groups is not relevant to the present study, because different experimental groups were not compared to each other and no biological conclusions were drawn.
Blinding	In all cases, manual annotation of datasets was completed prior to obtaining computational segmentation results.

Reporting for specific materials, systems and methods

We require information from authors about some types of materials, experimental systems and methods used in many studies. Here, indicate whether each material, system or method listed is relevant to your study. If you are not sure if a list item applies to your research, read the appropriate section before selecting a response.

Materials & experimental systems

n/a	Involved in the study	
x		Antibodies
x		Eukaryotic cell lines
x		Palaeontology and archaeology
x		Animals and other organisms
x		Human research participants
x		Clinical data
x		Dual use research of concern

Methods

n/a	Involved in the study	
x		ChIP-seq
x		Flow cytometry
x		MRI-based neuroimaging
